# An Integrative Variant Scoring Function for Finding Novel Genes Associated with Ovarian and Thyroid Cancer

**DOI:** 10.3390/ijerph23040420

**Published:** 2026-03-26

**Authors:** Amanda Bataycan, Omodolapo Nurudeen, Jonathon E. Mohl, Khodeza Begum Mitchell, Ming-Ying Leung

**Affiliations:** 1 Computational Science Program, The University of Texas at El Paso, El Paso, TX 79968, USA; ambataycan@miners.utep.edu (A.B.); jemohl@utep.edu (J.E.M.); 2 Bioinformatics Program, The University of Texas at El Paso, El Paso, TX 79968, USA; oinurudeen@miners.utep.edu; 3 Department of Mathematical Sciences, The University of Texas at El Paso, El Paso, TX 79968, USA; 4 Border Biomedical Research Center, The University of Texas at El Paso, El Paso, TX 79968, USA; kbegum@utep.edu; 5 Department of Biological Sciences, The University of Texas at El Paso, El Paso, TX 79968, USA

**Keywords:** ovarian cancer, thyroid cancer, single-nucleotide variants, functional effect analyzer, KEGG pathway analysis, type II diabetes mellitus, radioactive iodine treatment

## Abstract

**Highlights:**

**Public Health Relevance—How does this work relate to a public health issue?**

- Ovarian and thyroid cancers contribute to long-term disease burden that requires extended health management and survivorship care.
- This study analyzed genomics data in patients to identify novel genes likely associated with these two cancers and the results led to a hypothesis that their molecular pathways may both be related to type II diabetes mellitus, a global public health concern.

**Public Health Significance—Why is this work of significance to public health?**

- By applying an integrative gene-level variant scoring approach, this study identifies candidate pathways that may be jointly enriched across cancer and metabolic diseases.
- These findings generated a hypothesis about potential biological intersections between metabolic dysregulation and cancer that warrant further investigations.

**Public Health Implications—What are the key implications or messages for practitioners, policy makers and/or researchers in public health?**

- These results support further research examining metabolic and oncologic conditions in parallel when studying long-term population health.
- If validated in outcome-based studies, shared pathway signals could inform future strategies related to prevention research, risk stratification, and chronic disease management.

**Abstract:**

We devised a quantitative scoring function to assess the cumulative effects of somatic nonsynonymous single-nucleotide variants (SNVs) on protein-coding genes in patients with ovarian cancer (OvCa) and thyroid cancer (ThCa). The goal is to find novel candidate cancer-related genes for downstream bioinformatics analyses and wet-lab studies. With the Genomic Data Commons as primary data resource, SNV information was extracted from whole-exome sequencing data from patients with these cancers. A cumulative variant scoring function, *Q*(*G*), was developed to sum up the deleterious effects of the individual SNVs on gene *G*. While *Q*(*G*) can be computed using any popular functional effect analyzers such as FATHMM-XF, SIFT, PolyPhen, and CADD, we have also established an integrative scoring function *iQ*(*G*) that combines the deleterious assessments from different analyzers and demonstrated that *iQ*(*G*) is a more effective method for identifying likely cancer-related genes. Based on the *iQ*(*G*) rankings, the top three novel genes for OvCa are *AHNAK2*, *UNC13A*, and *PCDHB4;* and those for ThCa are *PLEC*, *HECTD4*, and *CES1*. Furthermore, the top 1% genes with highest *iQ*(*G*) scores for each cancer were submitted for KEGG pathway analysis. The results revealed that several genes of the *CACNA1* family within the type II diabetes mellitus pathway are likely related to both OvCa and ThCa and suggested other molecular interactions that should be further studied in connection with OvCa prognosis and ThCa treatment.

## 1. Introduction

Technological advancements over the past two decades have transformed biomedical research, enabling the integration of high-throughput sequencing technologies with computational biology. With next-generation sequencing (NGS), laboratories can efficiently generate large-scale genomic data from patient-derived samples. These data are often shared as machine-readable files containing information on genetic alterations such as single-nucleotide variants (SNVs). Computational tools are then used to organize, annotate, and analyze this information, allowing researchers to detect meaningful patterns across patient cohorts. A key objective of this study is to leverage these approaches in developing a quantitative scoring function to identify novel genes implicated in cancer by examining SNVs in tumor and normal tissue samples.

Improvements in cancer treatment have led to longer survival times for many patients. However, this progress has also revealed a subset of cancers with chronic behavior, characterized by periods of remission followed by recurrence. OvCa and ThCa are notable examples of such conditions that require long-term management, thus posing a significant challenge to public health. OvCa is one of the most lethal gynecological cancers, in part due to its asymptomatic early stages and lack of effective early detection methods. The American Cancer Society estimates that approximately 20,890 women in the United States were diagnosed with OvCa in 2025, and about 12,730 will die from the disease [1,2]. While early-stage detection can yield survival rates of 85–90%, the 5-year survival rate drops below 30% for advanced-stage cases [3]. ThCa is often detected at earlier stages, largely due to advances in imaging technologies such as CT scans and MRI. In 2025, an estimated 44,020 new cases were diagnosed in the U.S., with a higher prevalence among women and a relatively low mortality rate of about 5% [4,5]. Despite its generally favorable prognosis, ThCa can require long-term monitoring, particularly after surgical removal of the thyroid or radioactive iodine (RAI) therapy.

Cancer is essentially caused by DNA mutations, which can arise through endogenous mechanisms, such as errors during DNA replication, or from exogenous sources, including radiation and chemical exposure in the environment. One major consequence of such exposures is the production of reactive oxygen species (ROS), unstable molecules that disrupt cellular processes and compromise genomic stability. ROS can interfere with DNA replication and repair pathways, increasing the likelihood of mutagenesis [6,7].

Among various types of mutations, SNVs are particularly significant in cancer research. These involve a single base substitution within the DNA sequence. Both synonymous and nonsynonymous SNVs occur within protein-coding regions. Synonymous SNVs do not alter the amino acid sequence of the encoded protein, while nonsynonymous SNVs can lead to amino acid changes that may disrupt protein functions. Such alterations can affect the activities of genes that drive tumor formation, progression, or suppression, the disruption of which may impair key biological functions, ultimately leading to cancer.

Genes are composed of DNA segments that encode either a single transcript or multiple different transcript isoforms through alternative splicing or other mechanisms. According to transcriptomic studies, approximately 83% of human genes generate between 2 and 77 different transcripts [8]. This transcript diversity adds a layer of complexity when assessing the impact of mutations. The same SNV may affect multiple isoforms, and the functional consequences of that variant can vary across them. Transcript-level resolution is therefore essential in evaluating the potential deleterious effects of SNVs.

To evaluate the potential impact of SNVs on protein function, several bioinformatics tools have been developed. These functional effect analyzers include SIFT, PolyPhen, CADD, and FATHMM-XF, each using distinct computational strategies. SIFT and PolyPhen rely on sequence conservation and homology-based models while PolyPhen additionally incorporates structural information [9,10,11]. CADD uses integrated functional and conservation data to score variants by their likely deleteriousness [12]. FATHMM-XF employs machine learning techniques like hidden Markov models [13,14,15,16].

In this paper, we propose a scoring function that will integrate multiple functional effect analyzers to assess the cumulative effects of nonsynonymous SNVs on genes. In addition, system-level approaches such as Kyoto Encyclopedia of Genes and Genomes (KEGG) pathway analysis can be used to contextualize such effects. The STRING database [17,18,19,20,21,22,23,24,25,26,27,28,29], which supports an interface to conduct a variety of bioinformatics analysis, can be used to conduct these tasks and gain deeper insights into the biological roles of affected genes and their interrelationships.

## 2. Materials and Methods

Interpreting a large collection of NGS data can be computationally intensive, and integrating the data compiled from different sources is a delicate process that requires multiple steps but is necessary for the application of the proposed integrative scoring function. This methodology described herein ultimately led to predictions for both known cancer-related and novel genes, which then can be used for downstream bioinformatics analyses to assess functionalities at the genomic level.

### 2.1. Collecting, Organizing, and Extracting Information from Data Files

Data collection started with accessing the Genomic Data Commons (GDC) of the National Cancer Institute [30]. This public data sharing platform contains variant call format (VCF) files from The Cancer Genome Atlas [31] and other projects. All VCF files contain the basic mutation information, namely chromosome, position, reference sequence, and alternative sequence. A VCF file is structured into 3 different sections: metadata, header, and data. The metadata lines contain information including unique patient identification numbers, unique sample numbers, and, depending on the project, a description of the information included in the CSQ column of the data section. The CSQ information, which is gathered from both the Variant Effect Predictor (VEP) Version 88 and BioMart online tools in Ensembl, a bioinformatic project designed by EMBL-EBI & Wellcome Trust Sanger Institute, Cambridge, UK [32,33,34], contains a complete list of all potential genomic, transcriptomic, and functional effects based on the unique SNV. Due to the extremely condensed format of the provided CSQ entries per variant, this information in the original VCF files, when opened in either a text editor or Excel, is illegible. To overcome this problem, a Python code was written to decipher the CSQ contents and extract its corresponding mutational data and then convert them into columns in a dataframe (a 2-dimensional virtual table) with unique variants as the row entries and the columns containing their corresponding information.

The data section of each VCF file contains the somatic mutations in a pair of tumor and normal tissue samples from the same patient. The SNVs in the tumor and normal samples of the cohort of patients can be separated from each other, creating a tumor dataset and a normal dataset. In either dataset, the frequency of an SNV was recorded based on the number of patients that it is found in. If a patient had two different VCF files, and the same SNV was recorded in both files, it was considered a duplicate and was only counted once. If two different patients shared the same SNV, it was counted twice.

The VCF files of each of the two patient cohorts with OvCa and ThCa were then compiled into a single dataframe while simultaneously parsing the variants’ CSQ information into 72 separate columns based on the format described within the metadata; the list of CSQ entries can be found in the Supplementary File “CSQ Columns and Descriptions.xlsx”. The tumor and normal SNV frequencies among the different patients were merged onto this dataframe, which was used later.

Due to the different sources of cancer projects that uploaded data to the GDC platform, the CSQ information can vary among datasets, but the key information consistently included transcript IDs, genes, the region in which the transcript occurs, the mutational change type, the length of the transcripts, as well as SIFT and PolyPhen scores. With this information, we applied a filter to the compiled OvCa and ThCa dataframes to focus on only nonsynonymous variants in protein-coding genes, which were sent forward for subsequent analysis.

### 2.2. Functional Effect Analyzers

Working with the extracted mutational data, different scoring software can be applied to determine the functional effects or the deleteriousness of each variant. To incorporate multiple perspectives, four different analyzers were used for this study: (i) FATHMM-XF, (ii) CADD, (iii) SIFT, and (iv) PolyPhen. While SIFT and PolyPhen scores were already provided by the CSQ columns in the original VCF files, FATHMM-XF and CADD scores had to be obtained through SNPnexus [35,36,37,38,39] and the VEP tool in Ensembl [32,33] by submitting the basic information of chromosome (chrom), position (pos), reference sequence base (ref_seq), and the alternative (i.e., mutated) sequence base (alt_seq) of the SNVs. For this research and any future work requiring the usage of CADD scores, it is important to note when submitting the mutational data to VEP, the CADD feature needs to be enabled under the pathogenicity predictions in the additional configurations.

Input files in the formats required by these tools were generated by a Python code, and the returned outputs were merged onto the respective dataframes for the two patient cohorts. The Supplementary Files are named according to the information they contain, “X_FATHMM.csv” and “X_CADD.txt”, where X is either ThCa or OvCa. Since FATHMM-XF scores were based only on the variant information regardless of which transcript it is on, merging the rows, which each represent a unique SNV, is straightforward. In contrast, CADD scores assessed the SNV’s effects in the context of the transcript, necessitating the dataframe to be expanded based on the lists of possible transcripts found in the CSQ columns, thereby transforming each row to show the functional effect of the SNV on each unique transcript. In this format, the CADD scores were conditionally merged based on chrom, pos, ref_seq, alt_seq, and the unique transcript ID.

Table 1 contains the individual scoring ranges of the four analyzers, as well as their cutoff values with which they deem an SNV deleterious [9,10,11,12,13,14,15,16].

In view of the scoring and benign/deleterious variant classification differences among the four effect analyzers, we first apply the following min–max normalization to the scores to ensure that they are all within the range of 0 to 1:

(1) $$\mathrm{normalized}(x_{i})=\;\frac{x_{i}-x_{\min}}{x_{\max}-x_{\min}}$$

We also tried other normalization approaches, such as the z-score method, which produced different rankings of the genes, but the resulting collections of the top-ranking 1% genes were very similar to those obtained by min–max normalization. The simple min–max normalization was selected as it preserved relative differences while keeping all values nonnegative, which was desirable for the aggregation framework.

Furthermore, we replaced the SIFT scores, where lower scores indicate higher deleteriousness, with their complementary values (i.e., *x* is replaced by 1−x) so that higher scores uniformly indicate higher deleteriousness across all four effect analyzers. By filtering SNVs prior to min–max normalization, the $x_{\max}$ and $x_{\min}$ in Equation (1) become dependent on the selected deleterious cutoff, the scoring range of each effect analyzer, and the directionality of the inequality used for classification, as seen in Table 1. For example, for the FATHMM-XF analyzer, $x_{\max}$ would be 1, and $x_{\min}$ would be 0.5.

With the newly normalized scores, a quantitative scoring function *Q*(*G*), as displayed in Equation (2), can be applied to each effect analyzer and summarize the cumulative effects of the deleterious SNVs on a gene denoted by *G*. An SNV is considered deleterious if its original score is within the corresponding effect analyzers cutoff range and a gene is considered “pathogenic” if it contains at least one deleterious SNV. A *Q*(*G*) score can be computed for each pathogenic gene using Equation (2), where $N_{G}$ denotes the number of different transcripts of gene *G*.

(2) $$Q\left(G\right)=\frac{1}{N_{G}}\overset{N_{G}}{\underset{j=1}{\sum }}\frac{1}{ln\left(l\left(t_{j}\right)\right)}\underset{v\;\mathrm{in}\;t_{j}}{\sum }\mathrm{Score}\left(v\right)*[\mathrm{tumor}\left(v\right)-\mathrm{normal}\left(v\right)]$$

The rationale behind $Q\left(G\right)$ is that if a variant, *v*, is disruptive to the function of the gene, the functional effect analyzer would give it a high deleterious score, denoted by $\mathrm{Score}\left(v\right)$ in Equation (2). Furthermore, *v* would occur more frequently in tumor tissues than in normal tissues, leading to a large value for $\mathrm{Score}\left(v\right)*[\mathrm{tumor}\left(v\right)-\mathrm{normal}\left(v\right)]$, where $\mathrm{tumor}\left(v\right)$ and $\mathrm{normal}\left(v\right)$ respectively denote the numbers of patients with the variant v in their tumor and normal tissue samples. The inner sum in Equation (2) aggregates the cumulative effects of the individual SNV on the transcript $t_{j}$ (i.e., the *j*th transcript of gene *G*). As longer transcripts are naturally expected to have larger number of deleterious SNVs, this length effect is accounted for by the factor $1/(ln(l(t_{j})))$, where $l(t_{j})$ stands for the length of $t_{j}$. We chose to use $1/\ln\left(l(t_{j}\right))$ as the length penalty factor, as opposed to other possibilities like $1/l\left(t_{j}\right)\;\mathrm{or}\;1/\sqrt{l(t_{j})}$, following a similar analysis in another cancer dataset [40], which suggested that $1/\ln\left(l(t_{j}\right))$ led to the best performance of the scoring function. After completing this calculation for all the different transcripts of *G*, we take the average, which is accomplished by summing over all the transcripts and then dividing by $N_{G}$. Overall, genes associated with the cancer are expected to have high *Q*(*G*) scores.

### 2.3. The Integrative Scoring Function $iQ\left(G\right)$

The integrative variant scoring function, *iQ*(*G*), is developed to integrate the assessments of deleteriousness of SNVs by multiple functional effect analyzers. In Equation (3), an SNV is considered deleterious if it is classified so by at least one of the analyzers, where $\mathrm{AveScore}\left(v\right)$ is calculated by averaging the deleterious scores for *v* provided by all the effect analyzers being incorporated. This procedure allows us to take into consideration the individuality of the of all the effect analyzers’ prediction algorithms. Note that AveScore(*v*) is calculated as long as one of the effect analyzers has deemed the variant, *v*, deleterious, regardless of whether the other three effect analyzers consider it deleterious or not. For example, if only two effect analyzers have returned a score and only one found the variant deleterious, the average would still be taken between the two available scores. It was observed that approximately 5–10% of the scores were missing in the individual effect analyzers’ *Q*(*G*) cumulative scoring function compared to the AveScore(*v*) in *iQ*(*G*).

In this study, we integrated the four effect analyzers presented in Table 1.

(3) $$iQ\left(G\right)=\frac{1}{N_{G}}\overset{N_{G}}{\underset{j=1}{\sum }}\frac{1}{ln\left(l\left(t_{j}\right)\right)}\underset{v\;\mathrm{in}\;t_{j}}{\sum }\mathrm{AveScore}\left(v\right)*[\mathrm{tumor}\left(v\right)-\mathrm{normal}\left(v\right)]$$

Using any scoring function, one can rank all the pathogenic genes from 1 to *p*, where *p* denotes the number of pathogenic genes and the highest scoring gene is given rank 1. The code for computing the *Q*(*G*) and *iQ*(*G*) scores for the compiled OvCa and ThCa data were implemented in Python 3.9 and is available at www.github.com/bataycan/iQG_Analysis (accessed on 14 December 2025). The analysis pipeline can also be executed online at the website https://oncominer.utep.edu/iQG (accessed on 14 December 2025).

To assess the performance of *iQ*(*G*), we introduce here a measure called standardized average rank (SAR). When given any set of human genes, we can look up the ranks for each gene among the pathogenic genes. If a gene is not among the pathogenic genes, we will give them the rank of *p* + 1. Equation (4) displays the formula to calculate the SAR value for any given set of *k* human genes.

(4) $$SAR=\frac{\sum_{i=1}^{k}r_{i}}{k*p}-\frac{1}{p},\;\mathrm{where}\;r_{i}=\;\mathrm{rank}\;\mathrm{of}\;\mathrm{the}\;i\mathrm{th}\;\mathrm{gene}\;\mathrm{in}\;\mathrm{the}\;\mathrm{set}$$

The SAR, whose value is always a number between 0 and 1, allows us to assess the performance of *iQ*(*G*), where a lower SAR value for the list of already known cancer-related genes indicates better performance. A csv file of the known genes is provided within the above-mentioned GitHub repository (under branch iQG_Analysis_2026), along with an Excel worksheet to include the references to articles and databases from which the gene list was compiled.

We have compared the SAR value of *iQ*(*G*) against that of *Q*(*G*) calculated individually with CADD, FATHMM-XF, PolyPhen, and SIFT. In addition, the SAR values of the lists of known OvCa- and ThCa-related genes were compared against those of random gene sets of the same size as the known gene lists. Using Python, we repeatedly generated 1000 random sets comprising genes selected from the collection of 20,255 human protein-coding genes gathered from two sources: the HUGO Gene Nomenclature Committee and Gene Cards [41,42,43,44]. A *z*-test was then performed to statistically demonstrate that the SAR value of the list of known cancer-related genes was significantly lower than that of randomly selected sets of genes.

### 2.4. Bioinformatics Analyses

The two lists of genes with top 1% *iQ*(*G*) scores collected from the OvCa and ThCa cohorts were separately submitted to the STRING website (https://string-db.org) (accessed on 14 December 2025) to analyze their genomic functions. The bioinformatics analysis results, including protein–protein interactions, Gene Ontology terms and KEGG pathways, were automatically returned. We focused on the KEGG pathway results in this paper. For each pathway, a ‘strength’ column is given, which is a built-in statistical score assigned to determine whether the given set of genes is associated with the given pathway. The higher the score, the more likely that many of the genes submitted are connected to the pathway compared to the expected amount from a randomly selected set of genes [45,46,47]. Based on the strength scores, the top 12 pathways associated with the genes from the submitted set were identified. These results and their implications will be presented in the next section.

## 3. Results and Discussion

Once the data was compiled from the extracted VCF files for the two different cancer cohorts, a brief survey of the unique variant counts found between the normal and tumor samples were taken. With the patient information extracted from the original VCF files, it was observed that in several cases, a single patient was linked to two or more VCF files. Those duplicated files were merged so that they will not be double counted in the results.

### 3.1. Variant Summary Statistics

Table 2 shows the number of (i) VCF files extracted from GDC; (ii) unique patients, (iii) known cancer-related genes; (iv) unique SNVs categorized as occurring in normal tissues only, tumor only, and common to both; and (v) transcripts and (vi) genes containing SNVs for OvCa and ThCa. For unique SNVs, transcripts, and genes, two numbers are recorded separated by a forward slash. The first number represents the count prior to the filter application in which all SNVs are accounted for, while the second number is the count after only the nonsynonymous variants on protein-coding transcripts were selected.

In comparison, OvCa patients had over twice as many unique variants compared to the larger ThCa cohort and are distributed among a larger number of genes. In addition, the number of currently known OvCa-related genes is also almost two times that of ThCa. Note also that over 95% of the SNVs were found only in the tumor samples, whereas a very low percentage, 0.01–0.04%, occurred only in normal samples, and ~4% were seen in both.

**Figure 1:**
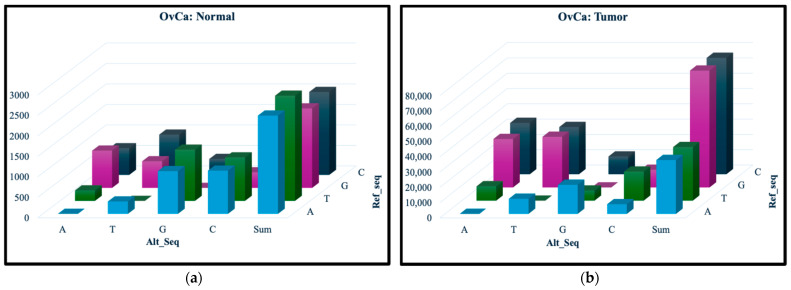
OvCa nucleotide change types in (**a**) normal and (**b**) tumor samples. The mutational change with the highest frequency in the normal sample was T > G at 1250 compared to the tumor sample’s C > A at 33,589.

**Figure 2:**
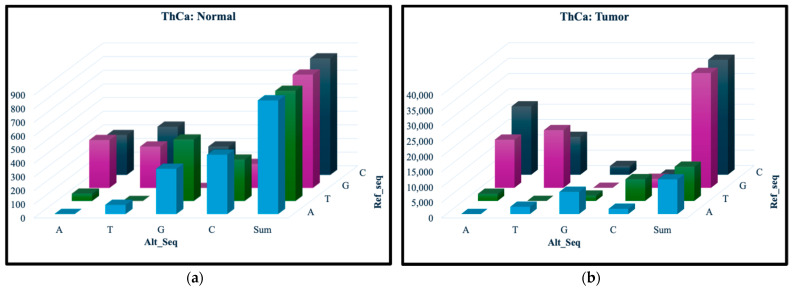
ThCa nucleotide change types in (**a**) normal and (**b**) tumor samples. The mutational change with the highest frequency in the normal sample was T > G at 450 compared to the tumor sample’s C > A at 22,288.

From the separate normal and tumor samples for both OvCa and ThCa, the SNVs can be classified based on their nucleotide change using the ref_seq and alt_seq columns in the dataframe. Figure 1 and Figure 2 show the occurrence of each of the 12 nucleotide change types, with the number of SNVs represented on the vertical axis, the ref_seq nucleotide along the diagonal and alt_seq nucleotide on the left–right axis. Comparing the vertical axes for the normal and tumor samples, we can see much fewer SNVs for all change types in the normal samples. Furthermore, the total number of the changes for each ref_seq nucleotide in the normal samples, as shown in the “Sum” columns in Figure 1a and Figure 2a, are quite similar. In contrast, the tumor samples (Figure 1b and Figure 2b) appear to have a much larger amount of G and C mutations, changing from the ref_seq G and C nucleotides to the alt_seq nucleotides A or T. These findings were observed for both OvCa and ThCa.

### 3.2. Assessing the Performance of iQ(G)

Table 3a shows the SAR values calculated for the two lists of known OvCa- and ThCa-related genes using the *Q*(*G*) rankings computed with the four individual functional effect analyzers. All SAR values are higher than those calculated for the same gene lists using *iQ*(*G*) rankings, as shown in Table 3b. It is therefore advantageous to use *iQ*(*G*) as a scoring function as it is more capable of giving superior rankings to the known cancer-related genes than the individual effect analyzers. Furthermore, since it is not guaranteed that every SNV inputted into an individual analyzer will receive a score, we frequently encountered the problem of missing scores for a portion of the SNVs when trying to calculate *Q*(*G*) with an individual analyzer. The use of *iQ*(*G*) helps minimize this problem because the combination of four analyzers reduces the chances of obtaining unscored SNVs (i.e., not scored by any of the analyzers) that must be left out of the gene scoring calculations. This is important for capturing mini-driver variants, which individually exert relatively low tumor-promoting impacts but collectively can have a substantial effect on cancer progression and persistence. The significance of mini-drivers in other cancer types is discussed in [48,49] and the references therein.

To confirm that *iQ*(*G*) can indeed effectively place the cancer-related genes at high rankings, we checked the SAR values for the lists of known OvCa- and ThCa-related genes against random gene sets sampled from all protein-coding genes in humans. The right column of Table 3b displays, for each cancer, the mean and standard error (SE) of the SAR values of 1000 randomly selected gene sets, each containing the same number of genes as the known cancer-related gene lists. For both OvCa and ThCa, the *z*-test shows that the mean SAR value of the random gene sets is significantly larger than that of the known gene list with *p*-value < 2.2 × 10^−16^, giving strong evidence for the effectiveness of *iQ*(*G*) as a scoring function to identify cancer-related genes.

The setup of the *iQ*(*G*) function allows it to be easily adapted to work with other alternative functional effect analyzers instead of, or in addition to, the four we have integrated in this study. Furthermore, the *iQ*(*G*) scoring, which currently takes the average of all possible transcripts of gene *G*, can also be refined in the future by using a weighted average of the transcripts to take their expression levels into account using transcriptomics data.

### 3.3. Genes with Top iQ(G) Scores in OvCa and ThCa

Table 4 lists the top 15 *iQ*(*G*) scored genes for OvCa and ThCa, where the genes highlighted in green are novel in the sense that they have not been associated with the respective cancer in the published literature.

The top three novel genes for each cancer type with their ranked position based on *iQ*(*G*) are presented in Table 5 along with brief notes on their known biological functions and disease involvement [50,51,52,53,54]. Given that these are novel genes for their related cancer, it was not surprising that only one, *AHNAK2*, was linked to another cancer while the rest were associated with other disorders and diseases.

Further investigation of the top novel gene *AHNAK2* suggested a hypothesis that could help explain the metastatic prognosis from late stage OvCa. From the 2022 article by Phung et al., although lung cancer spreading to the ovaries is a rare occurrence, it is not uncommon for ovarian cancer to metastasize to the lungs, which transpires in approximating 28.4% of patients [55]. As metastasized OvCa is generally found in late-stage untreated patients, mutations found within *AHNAK2* could also be a contributing factor to this outcome.

An interesting revelation in the ThCa patient group was the novel gene *CES1* and its relation to xenobiotics and drug metabolism, as noted in Table 5. RAI, which is a common treatment for ThCa, can be considered a xenobiotic. One study [56] found that while a single dose of RAI could be successful in treating ThCa for some patients, others needed several doses or complete thyroidectomy for treatment. Although the referenced study hypothesizes different factors like gender, age, thyroid hormone and autoantibody levels that affect the efficacy of RAI, an alternative perspective that has not been included is that the altered gene function of *CES1* is a potential culprit.

### 3.4. KEGG Pathway Analysis Results and Implications

**Figure 3:**
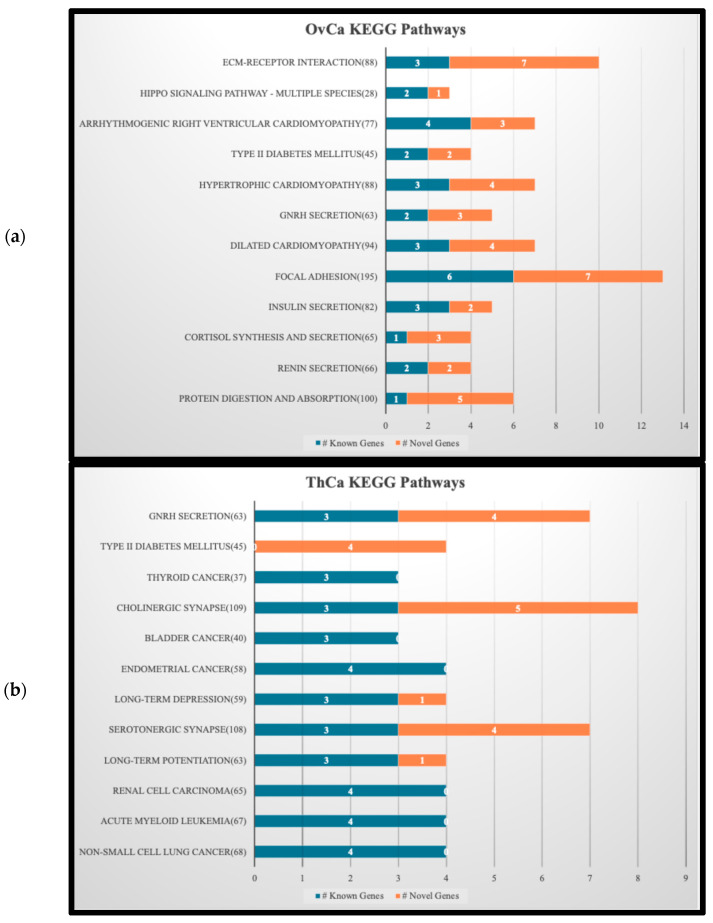
Top 12 KEGG pathways sorted by enrichment strength for (**a**) OvCa and (**b**) ThCa. Type II diabetes mellitus (DM2) appears at the 4th and 2nd spots for OvCa and ThCa, respectively.

The top 1% genes with highest *iQ*(*G*) scores, 149 from OvCa and 75 from ThCa, were selected for KEGG pathway analysis. Figure 3 is a stacked bar graph of the top 12 KEGG pathways found by submitting the selected genes according to the description in Section 2.4. In each pathway, the number of known genes related to the cancer is shown in blue and the number of novel ones in orange. The genes associated with these pathways, both known and novel, are listed on the Supplementary File “KEGG Bar Graphs.xlsx”. The background genes were not specified so STRING defaulted to all human protein-coding genes. In addition to the genes, this file also contains the full enrichment table for both OvCa and ThCa, including the strength and false discovery rate for each pathway.

**Figure 4:**
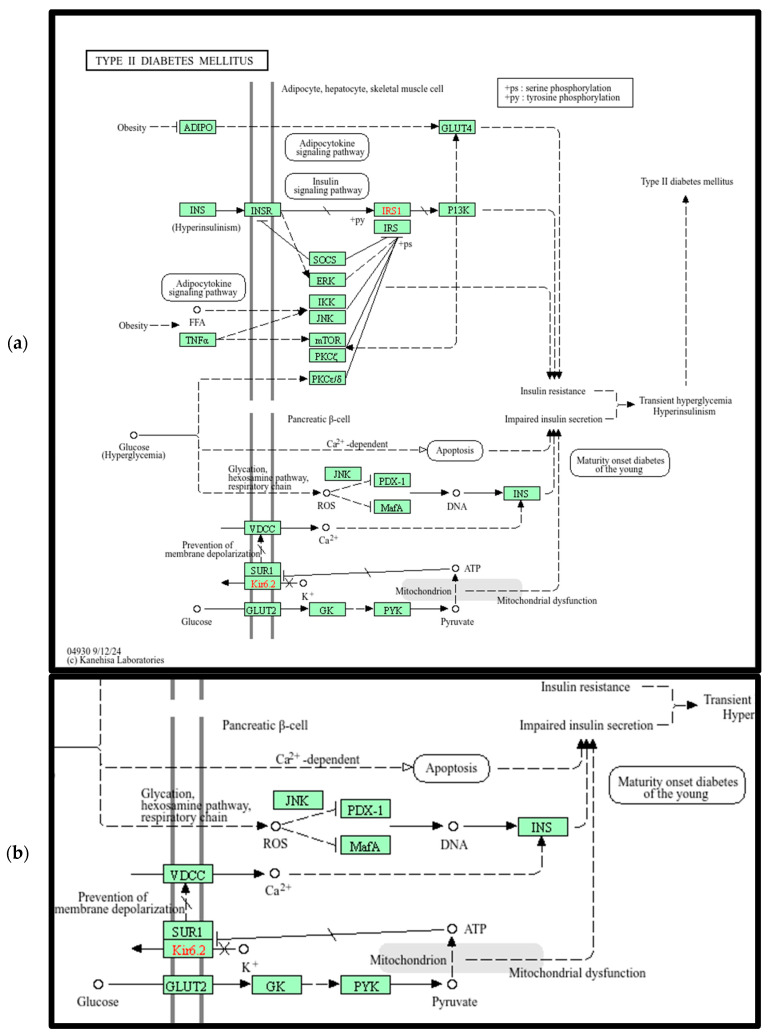
DM2 is a top KEGG pathway for OvCa and ThCa. (**a**) Full pathway diagram; (**b**) a cropped and zoomed-in view of the bottom left section of (**a**), illustrating potential genomic cascading effects associated with DM2. An interactive image can be viewed at the following link: https://www.kegg.jp/pathway/hsa04930 (accessed on 26 July 2024). As defined by KEGG, green boxes represent gene products, and red lettering indicates disease associated gene variants.

Figure 3a,b show that two pathways, namely DM2 and GNRH Secretion, are shared by OvCa and ThCa. We decided to perform a more in-depth analysis of the gene interactions within the DM2 pathway, as shown in Figure 4a, in relation to the two cancers. The four OvCa-related genes involved in the DM2 pathway are *CACNA1A*, *CACNA1C*, *CACNA1G* and *ABCC8*, while the four ThCa-related genes are *CACNA1A*, *CACNA1B*, *CACNA1C*, and *CACNA1G*. We will first discuss some possible roles of the *CACNA1* genes and then *ABCC8.*

*CACNA1A*, *CACNA1C*, and *CACNA1G* are among the top 1% genes based on *iQ*(*G*) score for both OvCa and ThCa. *CACNA1B* is among the top 1% for ThCa only but is within the top 5% for OvCa. All are members of the *CACNA1* gene family that encoded part of the voltage-dependent calcium channel (VDCC), which is embedded within the cell membrane and controls the flow of calcium ions. Following the pathway initiating from VDCC in Figure 4b, the release of calcium ions results in the expression of *INS*, which is responsible for insulin production, leading to impaired insulin secretion, hyperinsulinism, and finally DM2. The *CACNA1* gene family is already known to be related to OvCa [57,58], but no direct connection to ThCa has been reported to date. However, the study by Roh et al. (2021) found that patients with ThCa who underwent a thyroidectomy had an increased risk for DM2 and attributed the correlation to post-surgery synthetic thyroid hormone, age, gender or social habits [59]. Another study by Oberman et al. (2015) concluded that obesity and DM2 are significantly associated with differentiated ThCa [60]. Our findings have generated a potential hypothesis for future investigations: mutations in the *CACNA1* gene family, specifically in *CACNA1A*, *CACNA1B*, *CACNA1C*, and *CACNA1G*, that code for parts of the VDCC protein complex could be a possible connection between OvCa, ThCa, and DM2.

The *ABCC8* gene in the DM2 pathway is a known OvCa-related gene. It has been listed by Xiang et al. (2022) as one of 12 lactate metabolism-related genes that form a prognostic signature for OvCa; they established a prognostic scoring model where a lower expression level of *ABCC8* would lead to a higher risk score and poorer prognosis [61]. Since *ABCC8* encodes the SUR1 protein, which is a component of the ATP-sensitive potassium channel and serves as a sensory receptor to sense cellular energy, lower expression of *ABCC8* could repress production of the SUR1/Kir6.2 complex (see Figure 4b). This can trigger closure of potassium channels, which in turn causes over-expression of the VDCC complex to allow the flow of calcium ions, ultimately leading to DM2 via the same path described in the previous paragraph. So, for patients with OvCa who also suffer from DM2, *ABCC8* mutations could be a plausible reason behind the less favorable prognosis.

Also notable in Figure 3b is that five of the 12 pathways identified for our top-scoring ThCa genes are related to other cancers, one of which is acute myeloid leukemia (AML). RAI treatment, which is received by many ThCa patients, has been suspected to be a risk factor for AML [62]. However, based on Figure 3b, AML and ThCa share four genes, *NRAS*, *HRAS*, *BRAF*, and *AKT1*, that are known to be directly associated with them. This suggests an alternative hypothesis that SNVs on these four genes in patients with ThCa could also contribute to their susceptibility to AML as a secondary cancer. A more in-depth study on the roles of these genes in AML and ThCa would be worthwhile.

### 3.5. Study Limitations

While results of the current study have been used for generating some interesting hypotheses for future in-depth investigations, we should acknowledge that the public datasets we used to study OvCa and ThCa have certain limitations. First, not all loss-of-function variant types, which play important roles in disease pathogenesis, are included in these data. The only loss-of-function variant type identified within our SNV datasets were nonsense variants, in which protein translation is prematurely terminated. Information on frameshifting indels or large deletions is not available. Second, patient demographics were unavailable. Some frequently observed SNVs within certain subpopulations may also be recorded among these SNVs, introducing noise to the data. Yet, without demographic information, these SNVs cannot be removed. It is, however, reasonable to assume that such variants would likely be identified as benign by most functional effect analyzers or would be observed in both tumor and normal tissue samples at similar frequencies. In the former case, the quantity $\mathrm{Score}\left(v\right)$ in Equation (2) and $\mathrm{Ave}\mathrm{Score}\left(v\right)$ in Equation (3) will be small. In the latter case, the difference $\left[\mathrm{tumor}\left(v\right)-\mathrm{normal}\left(v\right)\right]$ in both equations will be small. So, their influence on the *iQ*(*G*) score should be minimal.

It should also be noted that although the current *iQ*(*G*) scoring function in Equation (3) can assess the deleterious effects of SNVs on each possible transcript individually, it took an unweighted average of these effects over all transcripts of gene *G*. Since different transcripts of a gene can be expressed at different levels, the *iQ*(*G*) score can be further refined to take expression levels into account when suitable datasets with matching SNV and transcriptomics data become available.

## 4. Conclusions

Utilizing the considerable amount of publicly available SNV data obtained from patients with OvCa and ThCa, the *iQ*(*G*) scoring function, which integrates the occurrence frequencies and cumulative functional effects of the SNVs averaged over different transcripts of a protein-coding gene *G*, has been demonstrated to be a useful quantitative method for identifying and ranking cancer-related genes. KEGG pathway analysis using the top-scoring genes found by *iQ*(*G*) for OvCa and ThCa revealed an interesting finding on how several members of the *CACNA1* gene family could be a possible link between these chronic cancers and DM2. The analysis also provided some insights into the prognosis and treatments for patients with OvCa and ThCa, which can be further investigated in the future.

## Figures and Tables

**Table 1:** Score ranges and deleterious cutoffs for the four effect analyzers [9,10,11,12,13,14,15,16].

	FATHMM-XF	CADD	SIFT	PolyPhen
Score Range	0–1	0–99	0–1	0–1
Deleterious Cutoff	≥0.5	≥10	≤0.05	≥0.447

**Table 2:** Summary counts for OvCa and ThCa datasets. The counts for unique SNVs, unique transcripts, and unique genes include an overall count for the whole dataset as well as a filtered count where only those with nonsynonymous SNVs in protein-coding regions are considered. The “/” separates the two counts.

	OvCa	ThCa
VCF Files	486	504
Unique Patients	462	496
Known Cancer-Related Genes	928	493
Unique SNVs	222,830/35,191	97,373/12,580
Normal only	78/6	7/0
Tumor only	213,894/34,794	94,051/12,374
Common	8858/391	3315/206
Unique Transcripts	56,240/45,759	193,512/25,083
Unique Genes	14,275/13,229	33,221/7507

**Table 3:** (**a**) SAR for known cancer-related genes ranked by *Q*(*G*) computed using individual functional effect analyzers. (**b**) SAR for known cancer-related genes ranked by *iQ*(*G*) along with the mean SAR and standard error (SE) of 1000 randomly selected gene lists ranked by *iQ*(*G*).

**(a)**				
	**FATHMM**	**CADD**	**SIFT**	**PolyPhen**
OvCa	0.6901	0.6234	0.6550	0.6584
ThCa	0.8516	0.7559	0.8242	0.8335
**(b)**				
	***iQ*(*G*)**	**Mean (± SE) for Randomly** **Sampled Human Gene Sets**		
OvCa	0.6018	0.6038 (±0.0003)		
ThCa	0.7527	0.8311 (±0.0005)		

**Table 4:** Top 15 *iQ*(*G*) scored genes in (**a**) OvCa and (**b**) ThCa. #Tr = number of transcripts and #Var = number of variants after filtering to keep only the SNVs on protein-coding genes. Genes highlighted in green have not yet been reported to be related to the respective cancer.

(a) OvCa					(b) ThCa			
Gene (*G)*	*iQ*(*G*)	#Tr	#Var		Gene (*G)*	*iQ*(*G*)	#Tr	#Var
*TP53*	24.16	23	135		*BRAF*	30.36	4	3
*TTN*	3.16	11	138		*NRAS*	4.09	1	2
*CSMD3*	2.77	4	36		*HRAS*	1.62	5	3
*HMCN1*	1.82	1	28		*TTN*	1.36	11	37
*HERC2*	1.74	1	24		*PLEC*	1.29	11	25
*AHNAK2*	1.67	1	34		*CLIP2*	1.22	2	7
*USH2A*	1.52	3	31		*CCAR1*	1.00	7	1
*UNC13A*	1.42	4	17		*HECTD4*	0.85	2	13
*CACNA1C*	1.37	23	19		*CES1*	0.79	3	2
*CSMD1*	1.36	7	20		*MUC4*	0.78	4	22
*RYR2*	1.35	5	34		*EPPK1*	0.76	2	14
*PCDHB4*	1.35	1	15		*EVPL*	0.75	2	8
*DNAH3*	1.32	1	21		*RPS18*	0.72	2	1
*MYH4*	1.29	1	17		*CACNA1C*	0.70	22	9
*DNAH10*	1.26	5	26		*TENM2*	0.68	3	9

**Table 5:** Top 3 identified novel genes for OvCa and ThCa; notes were extracted from references [50,51,52,53,54].

	Gene Name	Rank	Notes
OvCa	*AHNAK2*	6	Located on chromosome 14, this gene plays a role in calcium signaling; associated with non-small cell lung cancer.
	*UNC13A*	8	Located on chromosome 19, this gene is a part of the gene family that plays a role in neurotransmitter release at synapses; identified in amyotrophic lateral sclerosis.
	*PCDHB4*	12	Member of the protocadherin beta gene cluster on chromosome 5; highly suspected function includes specific cell–cell neural connections; mutations in this gene are linked to Seckel Syndrome and autism.
ThCa	*PLEC*	5	Located on chromosome 8, interlinks different elements on the cytoskeleton; mutations related to diseases including muscular dystrophy and epidermolysis bullosa.
	*HECTD4*	8	Located on chromosome 12, this gene is involved in glucose metabolic process and homeostasis; associated with neurodevelopment disorders and seizures.
	*CES1*	9	Responsible for hydrolysis or transesterification of xenobiotics (foreign synthetic chemicals); located on chromosome 16, alterations on this gene may affect drug metabolism.

## Data Availability

The data supporting the findings of this study are available on github at https://github.com/bataycan/iQG_Analysis (accessed on 14 December 2025). Original VCF files are accessible on the GDC data repository at https://portal.gdc.cancer.gov/analysis_page?app=Downloads (accessed on 14 December 2025).

## Supplementary Materials

The following supporting information can be downloaded at: https://www.mdpi.com/article/10.3390/ijerph23040420/s1, “CSQ Columns and Descriptions.xlsx”; “Compiled_KnownGenes.xlsx”; “KEGG Bar Graphs.xlsx”; “OvCa_CADD.csv”; “OvCa_FATHHM.csv”; “OvCa_KnownGenes.csv”; “OvCa_variants.csv.zip”; “ThCa_CADD.csv”; “ThCa_FATHHM.csv”; “ThCa_KnownGenes.csv”; “ThCa_variants.csv.zip”; iQG.py.

## Abbreviations

The following abbreviations are used in this manuscript:

OvCa	Ovarian cancer
ThCa	Thyroid cancer
SNV	Single-nucleotide variant
VCF	Variant call format
FATHMM-XF	Functional analysis through hidden Markov models—extended features
CADD	Combined annotation-dependent depletion
SIFT	Sort intolerant from tolerant
PolyPhen	Polymorphism phenotyping
VEP	Variant effect predictor
KEGG	Kyoto Encyclopedia of Genes and Genomes
STRING	Search Tool for the Retrieval of Interacting Genes/Proteins
NGS	Next-generation sequencing
GDC	Genomics Data Commons
CSQ	Consequence (data entry within VCF)
ROS	Reactive oxygen species
RAI	Radioactive iodine
AML	Acute myeloid leukemia
DM2	Type II diabetes mellitus
VDCC	Voltage-dependent calcium channel
